# Neutrophils in Bronchoalveolar Lavage Fluid Indicating the Severity and Relapse of Pulmonary Sarcoidosis

**DOI:** 10.3389/fmed.2021.787681

**Published:** 2022-02-02

**Authors:** Haoshen Feng, Lili Yan, Yabin Zhao, Zhenhua Li, Jian Kang

**Affiliations:** ^1^Department of Pulmonary and Critical Care Medicine, Shengjing Hospital of China Medical University, Shenyang, China; ^2^Department of Pulmonary and Critical Care Medicine, The First Affiliated Hospital of Jinzhou Medical University, Jinzhou, China; ^3^Department of Pulmonary and Critical Care Medicine, Institute of Respiratory Diseases, The First Affiliated Hospital of China Medical University, Shenyang, China

**Keywords:** pulmonary sarcoidosis, neutrophils, bronchoalveolar lavage fluid, severity, relapse

## Abstract

**Background:**

Pulmonary sarcoidosis is a highly heterogeneous granulomatous disease without any specific symptoms and manifestations. Neutrophils in bronchoalveolar lavage fluid (BALF) have been proposed to indicate the severity and prognosis of pulmonary sarcoidosis, but this needs confirmation in patients from different populations due to the heterogeneity of the disease. This study aimed to determine the characteristics of patients with pulmonary sarcoidosis in northeastern China and to explore the relationship between neutrophils in BALF and the severity of pulmonary sarcoidosis.

**Methods:**

We enrolled 432 patients who were diagnosed with pulmonary sarcoidosis in this retrospective study. The symptoms, extrapulmonary involvement, forced vital capacity percentage predicted (FVC % pred), and diffusing capacity of the lung for carbon monoxide percentage predicted (DLco % pred) were recorded. BAL was performed in 319 patients, and the results of a cellular examination of BALF were collected. A total of 123 patients who received corticosteroid treatment were followed up for at least 12 months, and the outcomes were recorded.

**Results:**

Cough was the most common symptom, and cutaneous involvement was the most common extrapulmonary manifestation in 304 (70.4%) and 82 (19.0%) patients, respectively. The percentages of patients with high neutrophil counts in BALF (>3%) were higher at Stages 2 and 3 compared with Stages 0 and 1 (33.2 vs. 19.4%, *p* = 0.007), although the percentages of neutrophils in BALF showed no difference between patients at Stages 0, 1, 2, and 3. Patients with high neutrophil counts in BALF had lower FVC % pred compared with the other patients (79.5 ± 18.2 vs. 84.9 ± 14.5%, *p* = 0.025) and were prone to relapse after corticosteroids were tapered. High neutrophil counts in BALF were independently associated with relapse after corticosteroids were tapered in a binary logistic regression analysis (*p* = 0.027).

**Conclusions:**

Patients with pulmonary sarcoidosis lacked specific symptoms and manifestations. The neutrophil count in BALF could indicate the severity and outcomes of pulmonary sarcoidosis.

## Introduction

Sarcoidosis is a systematic granulomatous disease with elusive etiology, which can manifest in almost any organ, such as the lungs, heart, skin, and eyes (1–3). The lungs are affected in more than 90% of cases of sarcoidosis, with a spectrum of manifestations ranging from enlargement of bilateral hilar lymph nodes to pulmonary fibrosis. The symptoms of pulmonary sarcoidosis are non-specific and the clinical course also leads to different outcomes (4, 5). Although some markers, such as serum angiotensin-converting enzyme, adenosine deaminase, CXCLs, YKL-40, and neopterin, indicate the activity of pulmonary sarcoidosis (6–9), specific markers that reflect the severity and prognosis of pulmonary sarcoidosis are still lacking.

The percentage of neutrophils in bronchoalveolar lavage fluid (BALF) has been proposed to reflect the severity and prognosis of pulmonary sarcoidosis in the past few decades. Researchers have found an increase in the neutrophil count in BALF from Stage 1 to Stage 3 in patients with pulmonary sarcoidosis (10), and high neutrophil counts in BALF indicate the occurrence of pulmonary diffusion dysfunction (11). It was also reported that patients with increased percentages of neutrophils in BALF had a high probability of receiving steroid therapy (12, 13), and the number of polymorphonuclear neutrophils in BALF helped to distinguish the outcome between remission and a more severe course of pulmonary sarcoidosis (11). However, the above studies were mainly conducted in western Europe almost two decades ago, and sarcoidosis is highly heterogeneous among different races and ethnic groups. Therefore, it is intriguing to clarify the role of BALF neutrophils in patients with pulmonary sarcoidosis in other parts of the world.

The primary objective of this study was to explore the relationship between neutrophils in BALF and the severity or outcome of pulmonary sarcoidosis, and the secondary objective was to determine the characteristics of patients with pulmonary sarcoidosis in Northeast China.

## Materials and Methods

### Patients and Study Design

This retrospective observational study was conducted in the First Affiliated Hospital of China Medical University. We enrolled 432 patients who were diagnosed with pulmonary sarcoidosis from 2000 to 2015. The sarcoidosis was diagnosed according to the European Respiratory Society (ERS)/American Thoracic Society (ATS)/World Association of Sarcoidosis and Other Granulomatous Disorders (WASOG) consensus statement (14). Patients with chronic airways disorders, active pulmonary infectious diseases, respiratory tract tumors, history of organic or mineral dust exposure history, and other significant comorbidities or medical history were excluded. The ages, sex, smoking status, and sarcoidosis-related symptoms (cough, dyspnea, fatigue, weight loss, chest tightness, and fever) were recorded at the point of diagnosis. The Scadding stages of sarcoidosis were made by a radiologist (15). The manifestations of extrapulmonary involvement were defined as follows: (1) ocular involvement of sarcoidosis was diagnosed by ophthalmologists; (2) cutaneous involvement was examined by a dermatologist; (3) peripheral lymph node involvement was defined by pathological examination; (4) cardiac involvement was defined according to electrocardiography and transthoracic echocardiography, and cardiologists helped us to exclude the possibility of other heart diseases, such as congenital heart disease and coronary heart disease; and (5) pleural involvement was defined by pleural thickening or effusion according to chest images. The study was approved by the Clinical Research Ethics Committee of the First Hospital of China Medical University.

### Cellular Examination in BALF

Bronchoalveolar lavage was performed in 319 patients during fiber optic bronchoscopy as described previously (11). After premedication by atropine and lidocaine, BAL was performed by standardized washing of the right middle lobe, lingula, or the area of greatest radiological abnormality with two aliquots of 50 ml sterile saline at 37°C. Each aliquot was retrieved with gentle manual aspiration. In our study, the retrieved percentages of BALF at 30–50% were considered adequate. The percentage of erythrocytes in BALF samples was <5%, which indicated that the sample was not a hemorrhagic one. BALF was filtered through a 70-mm pore filter to remove mucus, and then the cellular compounds were obtained by centrifugation (400 × *g* for 5 min at 4°C). The cytospin slides of cells in BALF were stained with May-Grunwald-Giemsa for cell differential count. For lymphocyte subpopulation analysis, cells were incubated with monoclonal antibodies for CD4 and CD8 that were conjugated with fluorescein isothiocyanate (FITC) or phycoerythrin (PE), and the BALF samples were examined by flow cytometry.

### Corticosteroid Treatment and Follow-Up

Among the 319 patients who performed BAL, 239 patients received corticosteroid treatment during follow-up, and the other patients were observed without corticosteroid treatment. The decision for corticosteroid therapy was made by an experienced specialist by reference to the radiographic stages, the severity of symptoms, pulmonary function tests, quality of life, and tolerability to corticosteroids. The initial dose of prednisolone was 0.5 mg/kg/day, and it was gradually reduced to 15 mg/day after 3 months. Patients were treated with prednisolone at 10–15 mg/day for 6–9 months, and the treatment was gradually withdrawn within 3–6 months. No patients received steroid-sparing agents in this study. The outcomes (improvement, deterioration, or relapse) of patients who were followed up for at least 12 months were recorded. Disease improvement and deterioration were determined by chest imaging, pulmonary function status, and clinical symptoms (for example dyspnea), and relapse was defined as the recurrence of disease after being totally under control (16).

### Pulmonary Function Test and Arterial Oxygen Pressure Measurement

Spirometry and diffusion capacity were measured using a Jaeger MasterScreen system (Hoechberg, Germany) by 2-well trained technicians according to American and European respiratory society guidelines (standardized lung function testing). The results of forced vital capacity (FVC), forced expiratory volume in the first second (FEV_1_), and diffusing capacity of the lung for carbon monoxide (DLco) were expressed as percentages of the predicted values (17). Arterial oxygen pressure (PaO_2_) was measured when patients were at rest without supplementary oxygen.

### Statistical Analysis

Data were expressed as the mean ± SD, and the data analyses were performed on SPSS for Windows version 22.0 (IBM Corporation, Armonk, NY, USA). Comparisons of measurement data were performed using Student's *t*-test and Mann-Whitney *U*-test based on whether data were in the normal distribution. Fisher's exact test was used to evaluate the differences in numerical data between groups. The association of parameters and relapse after corticosteroid tapering were assessed by multivariate binary logistic regression analysis. *p* < 0.05 was regarded as significant.

## Results

### Patients' Characteristics

In total, 432 patients in this study were included, i.e., 6 patients at stage 0, 155 patients at stage 1, 261 patients at stage 2, 10 patients at stage 3, and none at stage 4. Three hundred and sixty-five patients were diagnosed based on histological confirmation and the other 67 were diagnosed based on clinical characteristics. Among the patients diagnosed based on histology, 160 were diagnosed by bronchial mucosal biopsy and 70 by mediastinal lymph node biopsy via transbronchial needle aspiration or mediastinoscopy. Besides, 24 patients were diagnosed by lung tissue biopsy via thoracoscopy, percutaneous puncture, or thoracotomy. In addition, 50, 59, and 2 patients were diagnosed with superficial lymph node biopsy, cutaneous biopsy, and oscular biopsy, respectively.

The age, male/female ratio, smoking status, sarcoidosis-related symptoms, manifestations, FVC % pred, FEV_1_/FVC, DLco % pred, PaO_2_, BALF cells differential count, and the ratio of CD4/CD8 are listed in Table 1. Cough was present in 304 (70.4%) of these patients and was the most common symptom. Cutaneous involvement was the most common extrapulmonary manifestation and occurred in 82 (19%) patients. We compared these characteristics between patients at Stages 0 and 1 and Stages 2 and 3. Patients at Stages 2 and 3 were more likely to have pleural involvement (*p* = 0.039) compared with those at Stages 0 and 1, and there was no difference in symptoms between the two groups. DLco % pred (*p* = 0.033) and PaO_2_ (*p* = 0.005) were decreased more significantly in patients at Stages 2 and 3 than in patients at Stages 0 and 1. Although there was no significant difference in the percentages of BALF neutrophils between patients at Stages 0 and 1 and Stages 2 and 3, the percentage of patients with high neutrophil counts in BALF (>3%) was higher at Stages 2 and 3 compared with Stages 0 and 1 (33.2 vs. 19.4%, *p* = 0.007). The percentages of eosinophils in BALF were also significantly higher at Stages 2 and 3 than Stages 0 and 1 (0.48 ± 0.97% vs. 0.20 ± 0.51%, *p* = 0.034).

**Table 1:** Clinical and bronchoalveolar lavage fluid characteristics of patients with pulmonary sarcoidosis at Stages 0 and 1 and Stages 2 and 3.

	**Total patients**	**Stage 0&1**	**Stage 2&3**	***P*** **-value**
Number of patients	432	160	272	–
Age (years)	49.1 ± 9.4	48.5 ± 9.5	49.5 ± 9.3	0.298
Sex (female/male)	339/93	129/31	210/62	0.239
Smoker (current or former), *n* (%)	45 (10.4%)	14 (8.75%)	31 (11.4%)	0.242
FVC % pred	83.3 ± 15.3	85.5 ± 15.5	82.1 ± 15.1	0.077
FEV_1_/FVC	79.8 ± 12.4	81.3 ± 14.4	79.0 ± 11.1	0.120
DLco % pred	80.6 ± 18.4	84.3 ± 18.8	78.5 ± 18.0	0.033
PaO_2_ (mmHg)	81.4 ± 13.4	84.7 ± 14.8	79.8 ± 12.4	0.005
**Symptoms**, ***n*** **(%)**				
Cough	304 (70.4%)	108 (67.5%)	196 (72.1%)	0.253
Dyspnea	163 (37.7%)	52 (32.5%)	111 (48.8%)	0.052
Chest tightness	152 (35.2%)	55 (34.4%)	97 (35.7%)	0.435
Fatigue	103 (23.8%)	38 (23.8%)	65 (23.9%)	0.535
Weight loss	52 (12.0%)	15 (9.4%)	37 (13.6%)	0.124
Fever	46 (10.7%)	13 (8.1%)	33 (12.1%)	0.126
**Manifestations**, ***n*** **(%)**				
Cutaneous involvement	82 (19.0%)	31 (19.4%)	51 (18.8%)	0.484
Peripheral lymph node involvement	50 (11.6%)	19 (11.9%)	31 (11.4%)	0.498
Pleural involvement	67 (16.0%)	18 (11.3%)	49 (18.0 %)	0.039
Cardiac involvement	9 (2.1%)	1 (0.6%)	8 (2.9%)	0.095
Ocular involvement	5 (1.2%)	2 (1.3%)	3 (1.1%)	0.611
Number of patients receiving BAL, n (%)	319 (73.8%)	108 (67.5%)	211 (77.6%)	0.024
**BALF characteristics**				
Total cell count, 10^6^ cells/mL	0.20 ± 0.15	0.21 ± 0.16	0.20 ± 0.15	0.898
AM %	62.2 ± 18.5	64.3 ± 17.8	61.2 ± 18.7	0.160
Neut. %	5.2 ± 10.8	4.4 ± 9.3	5.6 ± 11.5	0.362
Lymph. %	31.9 ± 17.8	30.8 ± 17.2	32.4 ± 18.1	0.443
Eosin. %	0.34 ± 0.32	0.20 ± 0.51	0.48 ± 0.97	0.034
Neut. %>3%, *n* (%)	91 (28.5%)	21 (19.4%)	70 (33.2%)	0.007
Lymph. %>30%, *n* (%)	162 (50.8%)	52 (48.1%)	110 (52.1%)	0.317
CD4/CD8 ratio	3.9 ± 2.9	3.3 ± 2.7	3.5 ± 3.0	0.585

*Data are number (%) or mean ± SD. FVC, forced vital capacity; DLco, diffusing capacity of the lung for carbon monoxide; FEV_1_, forced expiratory volume in 1 s; PaO_2_, arterial oxygen pressure; BALF, bronchoalveolar lavage fluid; AM, alveolar macrophages; Lymph, lymphocytes; Neut, neutrophils; Eosin, eosinophils; % pred, percentage of the predicted value*.

### High Neutrophil Counts in BALF Indicated the Severity of Pulmonary Sarcoidosis

To explore the relationship between BALF neutrophils and the severity of pulmonary sarcoidosis, patients were divided into two groups according to their BALF neutrophil counts >3% or ≤ 3%. We compared the sarcoidosis-related symptoms, manifestations, neutrophil counts in blood, percentages of patients that received corticosteroid treatment, FVC % pred, FEV_1_/FVC, DLco % pred, and PaO_2_ between patients with BALF neutrophil counts >3% and ≤ 3% (Table 2). The neutrophil counts in blood showed no difference between the two groups (4.8 ± 1.3 vs. 4.9 ± 1.4, *p* = 0.54). FVC% present in patients with high neutrophil counts in BALF (>3%) was significantly lower than in those with normal neutrophil counts (≤3%) in BALF (79.5 ± 18.2% vs. 84.9 ± 14.5%, *p* = 0.025), but no significant difference was found in other aspects between the two groups. The results indicated that high neutrophil count in BALF might correlate with severity of restrictive ventilation, diffusion dysfunction, and radiographic staging in patients with pulmonary sarcoidosis.

**Table 2:** Clinical characteristics of patients with Neut. >3% and Neut. ≤3% in BALF.

	**Neut.>3%**	**Neut.≤3%**	***P*** **-value**
Number of patients	91	228	–
FVC% pred	79.5 ± 18.2	84.9 ± 14.5	0.025
FEV_1_/FVC	81.7 ± 10.2	79.7 ± 12.0	0.253
DLco% pred	79.5 ± 22.7	82.4 ± 17.5	0.401
PaO_2_ (mmHg)	81.0 ± 12.8	81.8 ± 13.2	0.691
Counts of Neut. in blood (× 10^9^/L)	4.8 ± 1.3	4.9 ± 1.4	0.540
Corticosteroids treatment, *n* (%)	71 (78.0%)	168 (73.7%)	0.476
**Symptoms**, ***n*** **(%)**			
Cough	67 (73.6%)	175 (76.8%)	0.302
Dyspnea	39 (42.9%)	105 (46.1%)	0.336
Chest tightness	40 (44.0%)	79 (34.6%)	0.082
Fatigue	18 (19.8%)	67 (29.4%)	0.049
Weight loss	9 (9.9%)	33 (14.5%)	0.179
Fever	10 (11.0%)	26 (11.4%)	0.540
**Manifestations**, ***n*** **(%)**			
Cutaneous involvement	16 (17.6%)	43 (18.9%)	0.457
Peripheral lymph node involvement	13 (14.3%)	32 (14.0%)	0.546
Pleural involvement	15 (16.5%)	39 (17.1%)	0.512
Cardiac involvement	3 (3.3%)	5 (2.2%)	0.413
Ocular involvement	0 (0%)	4 (1.8%)	0.258

*Data are number (%) or mean ± SD. FVC, forced vital capacity; DLco, diffusing capacity of the lung for carbon monoxide; FEV_1_, forced expiratory volume in 1 s; PaO_2_, arterial oxygen pressure; Neut., neutrophils; % pred, percentage of the predicted value*.

### High Neutrophil Count in BALF Indicated Relapse of Pulmonary Sarcoidosis After Corticosteroid Treatment

**Figure 1:**
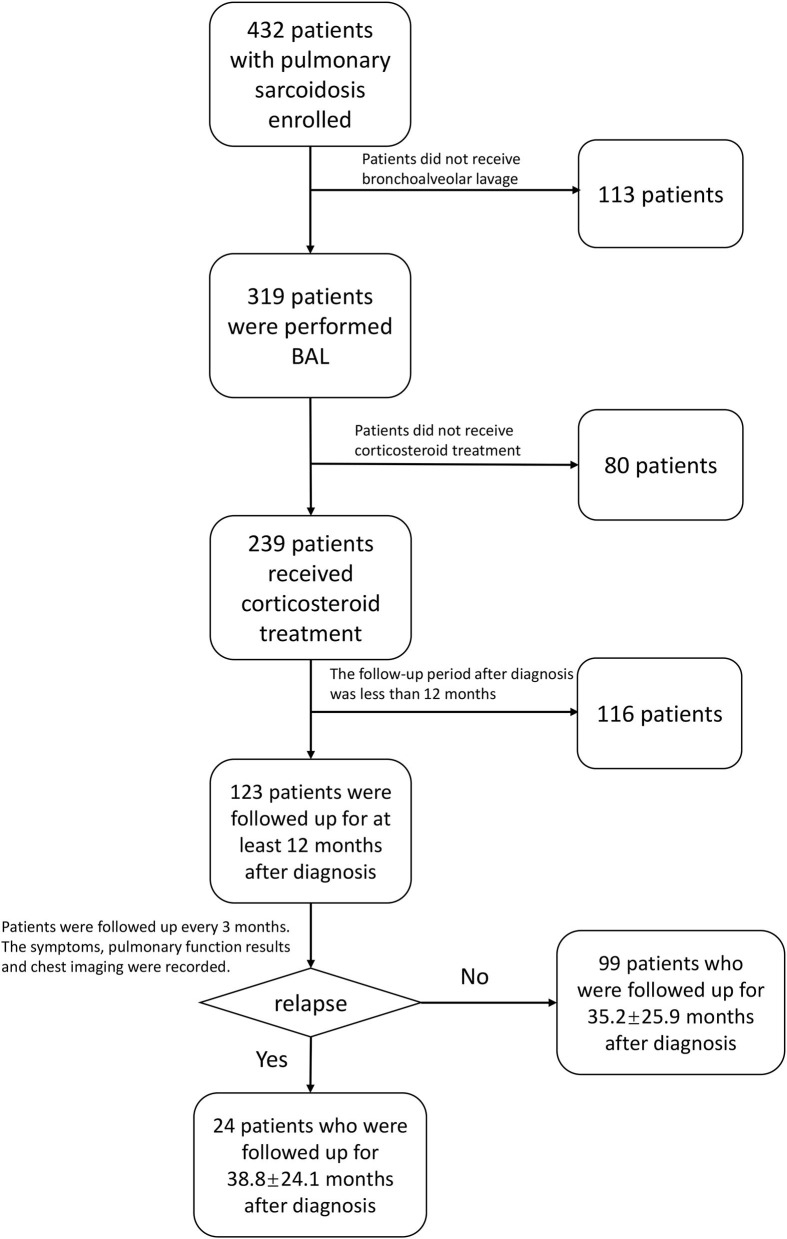
The flow diagram of the follow-up in this study. BAL, bronchoalveolar lavage.

A total of 123 patients (88 Stages 2 and 3 and 35 Stages 0 and 1) received corticosteroid treatment and were followed up for at least 12 months. The median duration of follow-up from diagnosis and from the stoppage of glucocorticoids was 29 and 14 months, respectively. Among patients in Stages 2 and 3, 32 patients experienced a change in thoracic staging from Stages 2 and 3 to Stages 0 and 1. All patients had the same thoracic staging at the time of recurrence compared with the initial diagnosis, and no patient was progressed to Stage 4 during our follow-up period. When corticosteroid treatment was tapered, 24 patients who relapsed during follow-up formed the relapsed group, and the other 99 patients formed the non-relapsed group. The flow diagram of follow-up in this study is shown in Figure 1.

We compared age, female/male ratio, duration of follow-up, the ratio of patients at Stages 2 and 3, FVC % pred, FEV_1_/FVC, DLco % pred, PaO_2_, and BALF cell differential count between the relapsed and non-relapsed patients (Table 3). The percentage of patients with high neutrophil counts in BALF (>3%) was significantly higher in the relapsed than non-relapsed group (50 vs. 26.3%, *p* = 0.024), although no difference was found in the percentages of BALF neutrophils between the two groups (5.8 ± 10.3% vs. 4.8 ± 10.2%, *p* = 0.632). In a univariate binary logistic regression analysis, high neutrophil counts in BALF (>3%; *p* = 0.027) were associated with relapse of pulmonary sarcoidosis after corticosteroid treatment, whereas other factors, such as age, sex, and lymphocyte count in BALF, were not (Table 4).

**Table 3:** Clinical and bronchoalveolar lavage fluid characteristics of patients with pulmonary sarcoidosis in relapsed group and non-relapsed group.

	**Relapsed group**	**Non-relapsed group**	***P*** **-value**
Number of patients	24	99	–
Sex, female/male	22/2	84/15	0.309
Age (years)	49.3 ± 11.3	49.1 ± 7.9	0.922
Patients in Stage 2&3, *n* (%)	18 (75%)	70 (70.1%)	0.443
Smoker (current or former), *n* (%)	2 (8.3%)	7 (70.7%)	0.556
Duration of follow-up (months)	38.8 ± 24.1	35.2 ± 25.9	0.521
FVC %, pred	80.9 ± 21.1	82.8 ± 14.4	0.736
FEV_1_/FVC	82.3 ± 11.3	79.1 ± 10.8	0.300
DLco % pred	85.6 ± 12.8	79.7 ± 20.7	0.225
PaO_2_ (mmHg)	85.8 ± 22.8	79.6 ± 11.4	0.319
**BALF characteristics**			
Total cell count, 10^6^ cells/mL	0.21 ± 0.22	0.21 ± 0.16	0.953
AM %	61.3 ± 21.1	59.5 ± 17.6	0.700
Neut. %	5.8 ± 10.3	4.8 ± 10.2	0.632
Lymph. %	32.2 ± 19.2	34.7 ± 17.0	0.556
Eosin. %	0.33 ± 0.70	0.42 ± 0.87	0.591
Neut. %>3%, *n* (%)	12 (50%)	26 (26.3%)	0.024
Lymph. %>30%, *n* (%)	12 (50%)	59 (59.6%)	0.319
CD4/CD8 ratio	3.6 ± 4.0	3.4 ± 2.2	0.265

*Data are number (%) or mean ± SD. FVC, forced vital capacity; DLco, diffusing capacity of the lung for carbon monoxide; FEV_1_, forced expiratory volume in 1 s; PaO_2_, arterial oxygen pressure; BALF, bronchoalveolar lavage fluid; AM, alveolar macrophages; Lymph., lymphocytes; Neut., neutrophils; Eosin., eosinophils; % pred, percentage of the predicted value*.

**Table 4:** Factors associated with relapse after corticosteroids tapered in a binary logistic regression analysis.

**Independent variable**	**Odds ratio (95% CI)**	***P*** **-value**
Age >40 years	0.497 (0.142–1.735)	0.273
Male	0.659 (0.131–3.321)	0.613
Smoker	1.034 (0.147–7.263)	0.973
Stage 2&3	1.206 (0.415–3.5)	0.731
Neut. %>3%	2.808 (1.122–7.023)	0.027
Lymph. %>30%	0.812 (0.318–2.076)	0.664

*CI, confidence interval; Neut., neutrophils; Lymph., lymphocytes*.

## Discussion

Our results showed that the percentages of patients with high neutrophil counts in BALF were increased in patients with pulmonary sarcoidosis at Stages 2 and 3 compared with those at Stages 0 and 1, and the high neutrophil counts in BALF might correlate with deterioration of pulmonary function and relapse after corticosteroid tapering. The present study helped to determine the characteristics of pulmonary sarcoidosis in northeastern China and confirmed the role of BALF neutrophils in pulmonary sarcoidosis based on previous studies.

Sarcoidosis is a systematic granulomatous disease without any specific symptoms and manifestations. The pathogenesis of this disease is currently thought to involve exposure of a genetically susceptible population to environmental antigenic stimulation and accumulation of immunocompetent cells in the lungs. Although lymphocytes and macrophages mainly participate in the formation of sarcoid granulomas, the role of BALF neutrophils in pulmonary sarcoidosis has gained more attention. Drent et al. first proposed that neutrophils in BALF might be considered as a marker of disease activity in pulmonary sarcoidosis (11). They found a significant increase in BALF polymorphonuclear neutrophils in patients who had a severe disease course compared with those in remission. The elevation of neutrophils level in BALF as the Scadding stage increased has been reported by several researchers (12, 13), and the increased neutrophil counts in BALF seemed to correlate with deterioration of pulmonary function, gas exchange dysfunction, and the need for steroid therapy (12, 18, 19). We also found that patients with high neutrophil counts in BALF were prone to restrictive ventilation, which was reflected by the decrease in FVC. Our results confirmed the probability that high neutrophil counts in BALF indicated the severity of restrictive ventilation in pulmonary sarcoidosis, and in our opinion, the effects of BALF neutrophils in pulmonary sarcoidosis might be due to the following mechanisms. First, neutrophils are reported to promote pulmonary fibrosis in interstitial lung diseases (ILDs), especially idiopathic pulmonary fibrosis, via the release of a series of profibrotic substances, such as neutrophil elastase and matrix metalloproteinases (MMPs) (20, 21). The degree of pulmonary fibrosis in high-resolution CT is also associated with neutrophil counts in BALF of patients with ILDs (22, 23). The increase in MMP-8 level in BALF leads to collagenase activity in patients with pulmonary sarcoidosis (24); therefore, we speculate that neutrophils might exert similar profibrotic effects in pulmonary sarcoidosis. Neutrophils might also participate in the formation and progression of granuloma. For example, neutrophil gelatinase-associated lipocalin (NGAL), which is a glycoprotein released from activated neutrophil granules and associated with the outcomes of pulmonary sarcoidosis (25), is reported to participate in granuloma formation by regulating lymphocytic inflammation during mycobacterial infection (26, 27). However, whether neutrophils promote the formation of granuloma through NGAL in pulmonary sarcoidosis remains to be explored in future studies. Besides, we did not find any relationship between diffusion dysfunction and neutrophil counts in BALF, which was not completely consistent with previous studies (11, 19). We speculate that the divergence might have been due to ethnic differences, which is worthy of further exploration.

Relapse of sarcoidosis in patients treated with corticosteroids occurs frequently when corticosteroids are tapered or discontinued. In a retrospective study, 74% of sarcoidosis patients who received corticosteroid therapy experienced relapse during a 4-year period, whereas only 8% of patients who were in spontaneous remission relapsed (28). The reason for relapse has not been clarified, although some risk factors have been found, such as smoking history and high level of circulatory CD45RO + Ki67 + T-regulatory cells (29, 30). Increment of neutrophils in the circulation and BALF has also been reported in relapsed patients (29, 31), but the mechanisms of neutrophils in the process of relapse remain elusive. In this study, 123 patients who received corticosteroid treatment were followed up for at least 12 months and 24 relapsed after corticosteroid tapering. The relapse rate (19.5%) in our study was lower than in a previous study, and we speculate this might be due to the following reasons: (1) follow-up duration of relapsed patients was 38.8 months in our study, which was shorter than in previous research; and (2) heterogeneity of the patient population. Consistent with previous studies, we found that neutrophil counts in BALF were increased in patients who were relapsed compared with the non-relapsed patients, and high neutrophil counts in BALF were a risk factor for relapse. In our opinion, second-line therapy (methotrexate, infliximab, etc.) should be started as soon as possible if patients in this subgroup are aggravated during corticosteroid tapering. Neutrophil inflammation seemed to indicate the poor outcomes after corticosteroid treatment in some pulmonary immune-related diseases. For example, neutrophil inflammation was associated with the disease severity and steroid resistance in patients with asthma (32, 33), and some researchers even proposed the alternative treatments to target neutrophils in asthma with a high neutrophil burden (34, 35). Although the pathogenesis of pulmonary sarcoidosis differs from that of asthma, we speculate that alternative antineutrophil treatment might be suitable for pulmonary sarcoidosis patients with high neutrophil counts in BALF in addition to corticosteroid treatment.

High neutrophil count in BALF might also indicate the occurrence of pulmonary infection. In this study, patients with potential infection were excluded when they were enrolled. First, patients with pneumonia-like pulmonary imaging manifestations or with increased infection-related biomarkers (for example procalcitonin) in serum were excluded. Besides, the pathogen culture of BAL was carried out in all patients who underwent fiber optic bronchoscopy, and patients with positive results were excluded. Finally, when pathogens were engulfed by macrophages or neutrophils during the cell differential counting of BALF, patients were also excluded. Thus, although the lack of PCR assay of BALF was a limitation of this study to some extent, patients with pulmonary infection were not included in this study. Another important consideration is that, although we found that high neutrophil counts in BALF were related to the severity of pulmonary sarcoidosis, no apparent neutrophil infiltration was found in the specimens obtained from a bronchial biopsy. In our opinion, sarcoid granulomas mainly consist of epithelioid cells, giant cells, macrophages, and lymphocytes (1–3), and the average percentage of neutrophils in BALF was only 5.2% in our study, which made it hard to observe neutrophil infiltration under the microscope.

There were some limitations to our study. First, although we found that high neutrophil counts in BALF indicated the severity and outcome of pulmonary sarcoidosis, the mechanisms of neutrophils in the pathogenesis were still unclear and need further exploration. Second, the percentages of eosinophils in BALF were increased in patients at Stages 2 and 3 compared with those in patients at Stages 0 and 1. Similar results were reported by other researchers and we speculate that the accumulation of eosinophils might be mediated by IP10 and Mig in lung tissues (11, 19, 36). However, we only focused on neutrophils in this study, and the effects of eosinophils in pulmonary sarcoidosis merit further study. Besides, cardiac involvement was diagnosed only by electrocardiography and transthoracic echocardiography in our study. The endocardial biopsy is the critical method to diagnose cardiac involvement in patients with sarcoidosis, whereas no patients underwent endocardial biopsy in this study. Finally, as this study was a retrospective, limited clinical manifestations and outcomes were acquired, especially in patients who relapsed.

## Conclusions

This retrospective study described the clinical characters of 432 patients with pulmonary sarcoidosis in northeastern China. Cough and cutaneous involvement were the most common symptoms and manifestations, and the percentages of patients with high neutrophil counts in BALF were increased in patients at Stages 2 and 3 compared with those in patients at Stages 0 and 1. Patients with high neutrophil counts in BALF (>3%) had lower FVC % pred at diagnosis than other patients had and were prone to relapse after corticosteroid tapering. Besides, high neutrophil counts in BALF were independently associated with relapse after corticosteroid tapering in binary logistic regression analysis. Based on previous studies, our results confirmed the probability that neutrophil counts in BALF indicated the severity and relapse of pulmonary sarcoidosis.

## Data Availability Statement

The raw data supporting the conclusions of this article will be made available by the authors, without undue reservation.

## Ethics Statement

The study was approved by the Clinical Research Ethics Committee of the First Hospital of China Medical University. The patients/participants provided their written informed consent to participate in this study.

## Author Contributions

YZ, ZL, and JK were involved in the conceptualization of the study. LY collected the data. HF and LY conducted the analysis. HF, LY, and YZ contributed to writing. All authors contributed to the article and approved the submitted version.

## Funding

This study was supported by grants from the National Key R&D Program of China (grant nos. 2016YFC0905700 and 2016YFC0901100).

## Conflict of Interest

The authors declare that the research was conducted in the absence of any commercial or financial relationships that could be construed as a potential conflict of interest.

## Publisher's Note

All claims expressed in this article are solely those of the authors and do not necessarily represent those of their affiliated organizations, or those of the publisher, the editors and the reviewers. Any product that may be evaluated in this article, or claim that may be made by its manufacturer, is not guaranteed or endorsed by the publisher.
